# Crosstalk between cancer-associated fibroblasts and non-neuroendocrine tumor cells in small cell lung cancer involves in glycolysis and antigen-presenting features

**DOI:** 10.1186/s10020-024-01051-y

**Published:** 2024-12-25

**Authors:** Yuanhua Lu, Hui Li, Peiyan Zhao, Xinyue Wang, Wenjun Shao, Yan Liu, Lin Tian, Rui Zhong, Haifeng Liu, Ying Cheng

**Affiliations:** 1 Postdoctoral Research Workstation, Jilin Cancer Hospital, Changchun, 130012 China; 2 Medical Oncology Translational Research Lab, Jilin Cancer Hospital, Changchun, 130012 China; 3 Jilin Cancer Hospital, Changchun, 130012 China; 4 Department of Medical Thoracic Oncology, Jilin Cancer Hospital, Changchun, 130012 China

**Keywords:** SCLC, CAF, Glycolysis, STING signaling, Antigen presentation

## Abstract

**Background:**

Small cell lung cancer (SCLC) is a highly fatal malignancy, the complex tumor microenvironment (TME) is a critical factor affecting SCLC progression. Cancer-associated fibroblasts (CAFs) are crucial components of TME, yet their role in SCLC and the underlying mechanisms during their interaction with SCLC cells remain to be determined.

**Methods:**

Microenvironmental cell components were estimated using transcriptome data from SCLC tissue available in public databases, analyzed with bioinformatic algorithms. A co-culture system comprising MRC5 fibroblasts and SCLC cell lines was constructed. RNA sequencing (RNA-seq) was performed on co-cultured and separately cultured MRC5 and H196 cells to identify differentially expressed genes (DEGs) and enriched signaling pathways. Glycolysis and STING signaling in SCLC cells were assessed using glucose uptake assays, qRT-PCR, and Western blot analysis. Immunohistochemical staining of SCLC tissue arrays quantified α-SMA, HLA-DRA and CD8 expression.

**Results:**

Non-neuroendocrine (non-NE) SCLC-derived CAFs exhibited more abundance and DEGs than NE SCLC-derived CAFs did, which interact with non-NE SCLC cells can induce the enrichment of glycolysis-related genes, increasement of glucose uptake, upregulation of glycolytic signaling proteins in non-NE SCLC cells and accumulation of lactate in the extracellular environment, confirming CAF-mediated glycolysis promotion. Additionally, glycolysis-induced ATP production activated STING signaling in non-NE SCLC cells, which upregulated T cell chemo-attractants. However, CAF abundance did not correlate with CD8 + T cell numbers in SCLC tissues. Additionally, non-NE SCLC cell-educated CAFs exhibited features of antigen-presenting CAFs (apCAFs), as indicated by the expression of major histocompatibility complex (MHC) molecules. Co-localization of HLA-DRA and α-SMA signals in SCLC tissues confirmed apCAF presence. The apCAFs and CD8 + T cells were co-located in the SCLC stroma, and there was a positive correlation between CAFs and regulatory T cell (Treg) abundance.

**Conclusion:**

Our findings suggest that crosstalk between CAFs and non-NE SCLC cells promotes glycolysis in non-NE SCLC cells, thereby increase T cell chemo-attractant expression via activating STING signaling. On the other hand, it promotes the presence of apCAFs, which probably contributes to CD8 + T cell trapping and Treg differentiation. This study emphasizes the pro-tumor function of CAFs in SCLC by promoting glycolysis and impairing T cell function, providing direction for the development of novel therapeutic approaches targeting CAF in SCLC.

**Supplementary Information:**

The online version contains supplementary material available at 10.1186/s10020-024-01051-y.

## Introduction

Small cell lung cancer (SCLC) is known as rapid growth, early metastasis and refractory (Rudin et al. 2021). Majority of SCLC patients were diagnosed as extensive stage and quickly develop resistance to chemotherapy and radiotherapy (Nicholson et al. 2016). Although combining chemotherapy with immune checkpoint inhibitors (ICIs) has marked a milestone in SCLC treatment, the survival benefits are limited (Cheng et al. 2022; Liu et al. 2021). The complex tumor microenvironment (TME) of SCLC is a critical factor in limiting therapeutic response, while our understanding of the interactions within the SCLC TME is still incomplete.

SCLC is a heterogeneous tumor, comprising both neuroendocrine (NE) and non-neuroendocrine (non-NE) cells, further classified into four subtypes (SCLC-A, SCLC-N, SCLC-P, SCLC-Y) based on the expression of lineage-defining transcription factors ASCL1, NEUROD1, POU2F3, and YAP1(Rudin et al. 2019). Additionally, there is a specific SCLC-I subtype with low expression of these transcription factors but high expression of inflammatory genes (Gay et al. 2021). Immune TME heterogeneity, elucidated through immunohistochemistry and single-cell transcriptomics, indicates that non-NE subtypes (SCLC-Y and SCLC-I) have more immune cell infiltration and inflammatory gene signatures than NE subtypes (SCLC-A and SCLC-N)(Dora et al. 2020; Tian et al. 2022). Recent studies have highlighted the crucial role of cancer-associated fibroblasts (CAFs) in SCLC TME heterogeneity, with hybrid-NE subtypes exhibiting more CAFs (Desai et al. 2024). Our previous work also demonstrated that CAFs promote NE SCLC cells reprogram to non-NE cells (Lu et al. 2024). According to these studies, CAF and immune features vary among different subtypes in SCLC.

In other cancers, the interaction between CAFs and tumor or immune cells has been well-documented. CAFs contribute to tumor cell proliferation, metabolic reprogramming, drug resistance, and metastasis(Chen et al. 2021). Metabolic reprogramming is hallmark of cancer, cancer cells rely primarily on glycolysis for glucose metabolism even in the presence of oxygen, which has been referred as the Warburg effect (Hsu et al. 2008). CAFs contact with cancer cells can lead to profound changes in glucose metabolism. Cancer cells can induce a glycolytic phenotype in CAFs, increasing their glucose uptake and metabolites efflux (Becker et al. 2020; Cruz-Bermúdez et al. 2019; Radhakrishnan et al. 2019). The metabolites, including lactate, glutamine and ketone bodies, are exploited by cancer cells, leading to a metabolic switch from glycolysis to respiration (Li et al. 2021). This process involves upregulation of lactate export transporter MCT4 in CAFs and lactate import transporter MCT1 in cancer cells, contributing to TME acidification, which impacts anti-tumor immune responses and drug efficacy(Apostolova et al. 2022; Payen et al. 2020). Notably, there are other ways to reshape tumor metabolism by CAFs, like the cargos of extracellular vesicle (EV), deriving from CAFs, can involve in metabolism reprogramming of tumor cells, even to promote glycolysis(Zhang et al. 2024; Zhao et al. 2016).

The interaction between CAFs and immune cells also plays a significant role in tumor progression. Certain CAF populations recruit myeloid-derived suppressor cells (MDSCs)(Yang et al. 2016; Zhu et al. 2023), exclude T cells (Grout et al. 2022), or promote M2 macrophage polarization (Chen et al. 2024), inducing immunosuppression. Noteworthy, Some CAF subtypes have direct immune-modulatory capacities, such as antigen-presenting CAFs (apCAFs) that express MHC class II genes and CD74, presenting antigens to CD4 + T cell in pancreatic ductal adenocarcinoma (Elyada et al. 2019a, b). The apCAFs also been found in other cancers including non-small cell lung cancer, breast cancer and colorectal cancer(Harryvan et al. 2022a, b; Kerdidani et al. 2022; Sebastian et al. 2020). However, apCAFs lack co-stimulatory molecules necessary for T cell activation and proliferation. Thus, apCAFs have been found to induce the naïve CD4 + T cells to regulatory T cells (TregsElyada et al. 2019a, b; Huang et al. 2022). Similar findings have been reported in colorectal cancer, where CAFs express MHC I molecules and cross-present antigens to CD8 + T cells but suppress their function due to the absence of co-stimulatory molecules (Harryvan et al. 2022a, b), thus the cytotoxic T cells might be trapped by cross-presenting CAFs in the stoma by antigen-specific manner. SCLC is not the stroma-rich cancer, whether CAFs from SCLC can regulate immune response by these manners are still unknown.

Here, we show that the crosstalk between CAFs and non-NE SCLC cells promotes glycolysis, activating STING signaling in non-NE SCLC cells and leading to the expression of T cell chemo-attractants, but CAFs has no impact on CD8 + T cell numbers. Additionally, our findings reveal the presence of apCAFs during the crosstalk between CAFs and non-NE SCLCcells, with CD8 + T cells and apCAFs co-localized in stromal regions. CAF abundance is positively correlated with Tregs, indicating a potential role for apCAFs in impairing T cell function. Thus, the interaction between CAFs and non-NE SCLC cells involves glucose metabolic reprogramming and T cell function, targeting these mechanisms may improve therapeutic response of SCLC.

## Materials and methods

### Cell culture

Human SCLC cell lines SBC-5, H196 and primary human lung fibroblast MRC-5 were purchased from Zhong Qiao Xin Zhou Biotechnology Company (Shang Hai, China), SCLC cell line H69 were obtained from Procell Life Science &Technology company. SCLC cell lines and MRC5 fibroblasts were cultured in RPMI 1640 and MEM medium, respectively, all of which were supplemented with 10% (*v/v*) FBS and 100 IU/mL of penicillin and streptomycin. Cells were incubated at 37 ℃ in a 5% CO_2_ in air humidified incubator. To construct the co-culture system, SCLC cells and MRC-5 cells were plated in 6-well plate or polyester membrane of transwell insert with 0.4-µm pore size (Labselect, Hefei, China) at a density of 1.5 × 10^5^/well, respectively. Cells act as inducers were seeded in polyester membrane of transwell, induced cells were seeded into 6-well plate.

### Transfection of siRNAs

Silencer Select siRNAs for human HK1, FGF5 and FGF17 were obtained from GenePharma (Suzhou, Zhejiang, China). SBC-5 and H196 cells were transfected with siRNAs for HK1, MRC5 fibroblasts were transfected with siRNAs for FGF5 and FGF7 by Lipofectamine RNAi MAX reagent (Invitrogen, Carlsbad, CA, USA) in accordance with the manufacturer’s instructions. SiRNA for Negative Control was used as the scrambled control.

### RNA-sequencing (RNA-seq) and pathway enrichment analysis

Total RNA of MRC-5 cells co-cultured with H69 or H196 or mono-cultured for 7 days, and H196 cells co-cultured with MRC-5 or mono-cultured for 7 days was extracted using TRIzol reagent, RNA-seq was performed by BGI (Wuhan, China). Three parallel replicates were prepared for each group. In brief, mRNAs were enriched by oligo(dT) magnetic beads, enriched mRNAs were fragmented and synthesized to cDNA. cDNA fragments were end repaired and adenylated at 3′ ends, then universal adapters were ligated to cDNA fragments, followed by PCR amplification. The library products were sequenced by BGIseq-500. Raw data were filtered using SOAPnuke (v1.5.6) and clean reads were aligned to the reference genome (GCF_000001405.39_GRCh38.p13) by HISAT. Then Bowtie2 was used to align clean reads to the reference gene sequence and obtained alignment results. The pathway analysis for differentially expressed genes (DEGs) was performed on the Dr. Tom network platform of BGI (http://biosys.bgi.com/).

### Datasets and bioinformatics analysis

The transcriptomic data of SCLC tissues were downloaded from cBioPortal for Cancer Genomics database (https://www.cbioportal.org/) (George_2015) and Gene Expression Omnibus database (https://www.ncbi.nlm.nih.gov/) (GSE60052) according to the guidance. Bioinformatics analysis was performed using R Studio (version 4.3.2). Immune and stroma cells were estimated using xCell (version 1.1.0), MCP-counter (version 1.2.0) and EPIC (version 1.1.7) packages which obtained from GitHub (https://github.com/). NE score was calculated as Zhang and colleagues reported (Zhang et al. 2018), Pearson correlations between expression of NE and non-NE genes in the George’ cohort or in the GSE60052 dataset was performed, NE score for every case was calculated by the formula: NE score = (correl NE − correl non-NE)/2. NE score > 0 referred as NE phenotype and NE score < 0 inferred as non-NE phenotype.

### RNA extraction and qPCR

Total RNA of cells was extracted using TRIzol reagents (Invitrogen, Carlsbad, CA, USA) and quantified by NanoDrop, followed by reverse transcription into cDNA using ReverTra Ace qPCR RT Master Mix (TOBOYO, Osaka, Japan). Real-time qPCR was performed using BeyoFast SYBR Green qPCR Mix and run on Agilent Mx3000P qPCR System (Palo Alto, CA, USA). 2^−ΔΔCt^ method was used to calculate comparative Ct and normalized to*β-actin*. Details of specific premiers used in this study were listed in Supplementary information.

### Western blotting

Total cell lysate was extracted using RIPA reagent complemented with Phosphatase and protease inhibitor mix (Beyotime, Beijing, China). Protein in cell lysate was quantified by BCA assay kit (Beyotime, Beijing, China) and equal amounts of protein of each group was resolved on 4–12% SDS-PAGE (ACE, Jiangsu, China) followed by transferred onto PVDF membranes (Millipore, Darmstadt, Germany). Blots were blocked with 5% milk at room temperature for 1 h and then cut prior to hybridization with primary antibodies at 4 ℃ overnight. Blots washed with TBST before HRP-conjugated secondary antibodies incubation. Chemiluminescent weas stimulated by ECL reagent (Beyotime, Beijing, China) and detected on GelDoc XRS + IMAGELAB system (Bio-Rad, CA, USA). Details of antibodies were listed in Supplementary information. Densitometric analyses of blots were performed using Image J software.

### Glucose uptake assay

Glucose uptake was measured using 2-NBDG probe. In brief, Co-cultured or individually cultured H196 or SBC-5 cells plated into 6-well plates at 1.5 × 10^5^ cells/well and cultured overnight, then cells were washed and incubated in medium with 50 µM 2-NBDG for 30 min at 37℃. After that, cells were washed twice and trypsinized, the intracellular 2-NBDG uptake was measured by detecting FITC fluorescence using a FACSCanto II flow cytometry.

### Lactate assessment

Lactate concentration in cell culture media was assessed using L-Lactic Acid (LA) Colorimetric Assay Kit (Elabscience, Wuhan, Hubei, China). In brief, SBC-5, H196 cells and MRC-5 fibroblasts which have undergone indicate treatment were co-cultured or cultured separately. Forty-eight hours before the end of the experiment, cell culture media was replaced with fresh one. Media was collected on the 7th day and centrifuged to remove sediment. Mixed the media of separately cultured SCLC cells and MRC5 cells evenly in equal proportions as control (the “Mo” group). The lactate concentration in media were detected according to the manufacturer’s instructions. All the lactate concentration was normalized by cell protein concentration.

### ELISA

Human FGF17 ELISA kit (COIBO BIO, Shanghai, China) was used to measure the FGF17 levels in culture media of MRC5 cells. Briefly, MRC5 fibroblasts were cultured separately or co-cultured with SBC-5 or H196 cells for 7 days, then SCLC cells were removed and cell culture media were replaced with fresh one. Forty-eight hours later, FGF17 levels in culture media were determined using ELISA kit according to the manufacturer’s instructions.

### ATP measurement

Intracellular ATP was measured by an ATP Luminescent Cell Viability Assay Kit (YEASEN, Shanghai, China). Briefly, co-cultured or individually cultured H196 or SBC-5 cells plated into 96-well plates at 1 × 10^4^ cells/well and cultured overnight, then remove the culture medium and washed cells by PBS, testing reagent was added to cells at 100 µl/well and mixed on the shaker at room temperature for 2 min. After placed for 10 min, the ATP luminescence signals were measured using a microplate reader (CLARIO star, BMG LABTECH, Offenburg, Germany) at 560 nm.

### Tissue arrays and immunohistochemical staining (IHC)

Tissue arrays (R801001) were obtained from ZK biotech company (Xi’an Shanxi, China), containing 80 samples from 37 SCLC patients and 3 healthy controls (Supplementary Table S1). IHC stains were performed on tissue arrays using ultra-sensitive SP IHC Kit (MXB biotechnologies, Fuzhou, Fujian, China). In brief, consecutive slides were rehydrated, then 3% H_2_O_2_ was used to block peroxidase activity. After that, antigen was retrieved using EDTA. Consecutive sections were subjected to staining with the following antibodies: α-SMA (Abclonal, 1:50, Wuhan, Hubei, China), CD8 (Proteintech, 1:20000, Chicago, Illinois, USA) and HLA-DRA (Proteintech, 1:800, Chicago, Illinois, USA), CD56 (Proteintech, 1:6000, Chicago, IL, USA) and REST (Proteintech, 1:400, Chicago, IL, USA). The stained slides were scanned using PanoBrain analyzer (Wuhan, Hubei, China). IHC score of α-SMA was evaluated as previous reported(Li et al. 2019), calculating by the proportion of positively cells per the whole cells (0, < 5%; 1, 6–25%; 2, 26–50%; 3, 51–75%; 4, 76–100%)(a) multiplied the intensity of staining (0, negative; 1, weak staining; 2, medium staining; 3, strong staining) (b). The CD8 + T cells density was measured by Aipathwell software(Benonisson et al. 2019), calculating by positive cell count/area of testing tissue.

### Statistics

Statistical analysis was performed using GraphPad Prism 8.0 software. All data were presented as mean ± SEM. Significance was measured using two tailed Student’s *t*- test or Mann–Whitney U test. Correlation analysis was determined using Spearman’s correlation. *P* value < 0.05 considered statistically significant.

## Results and analysis

### Heterogeneity of SCLC-derived CAFs in abundance and transcriptome

**Fig. 1:**
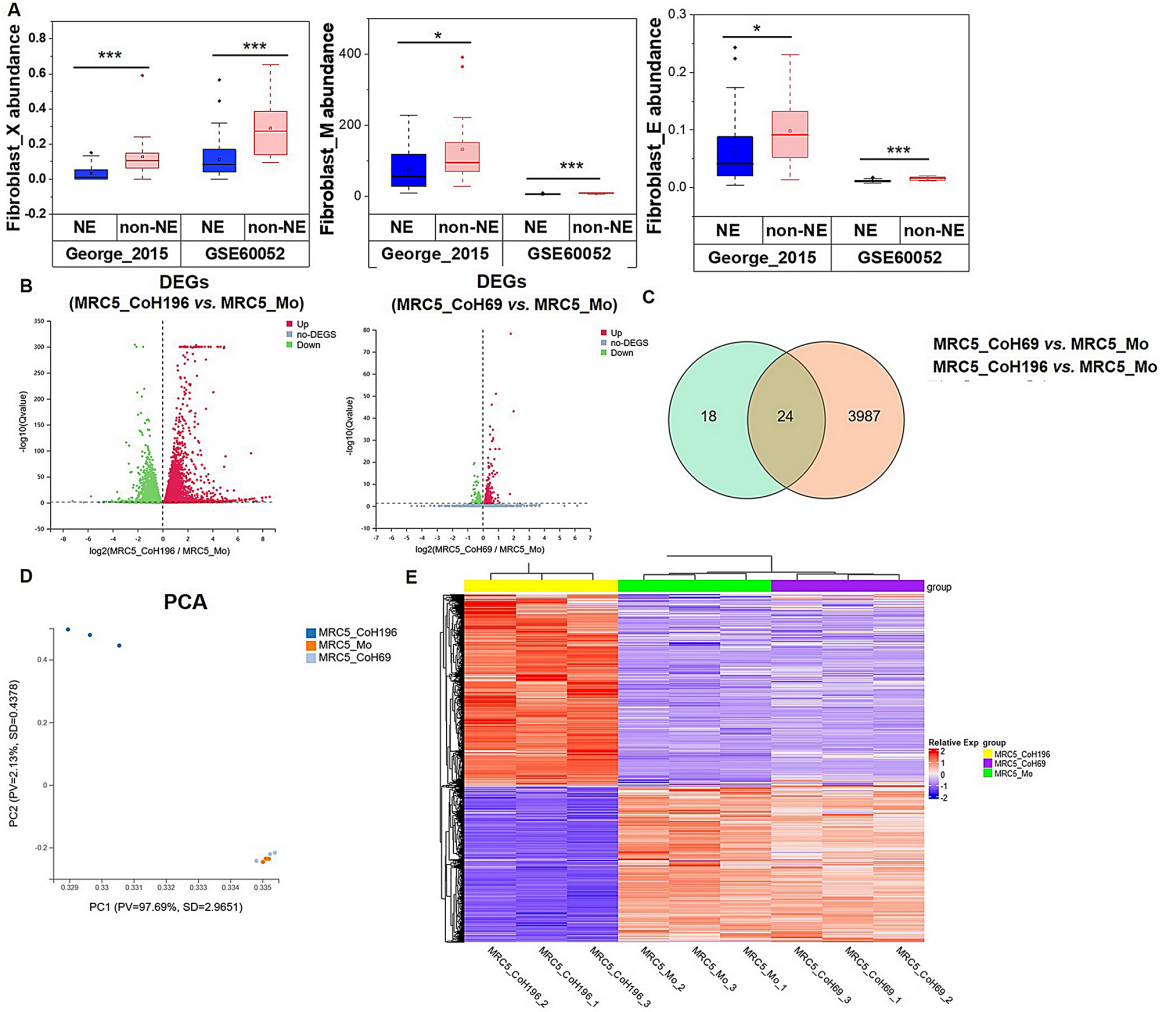
CAF abundance and expression profile heterogeneity in SCLC. (**A**) The comparison of CAF abundance from George’s cohort and GSE60052 dataset estimated by xCell (X), MCP-counter (M) and EPIC (**E**) algorithms between NE and non-NE subtypes. Mann–Whitney U test was used for determine significant difference, *P < 0.05, **P < 0.01, ***P < 0.001. (**B**) Volcano plot of DEGs between MRC5_coH196 and MRC5_Mo cells and DEGs between MRC5_coH69 and MRC5_Mo cells. (**C**) Venn diagram of DEGs between MRC5_coH196 and MRC5_Mo cells and DEGs between MRC5_coH69 and MRC5_Mo cells (Log2 Fold Change > 0.58). (**D**) PCA analysis for samples of MRC5_Mo, MRC5_coH69 and MRC5_coH196 cells. (**E**) Heatmap presents gene expression of MRC5_Mo, MRC5_coH69 and MRC5_coH196 cells

To explore the role of CAF in SCLC, we estimated CAF abundance in SCLC tissues using data from George’ study and GSE60052 dataset with xCell, MCP-counter and EPIC algorithms. Our analysis revealed that CAFs were more abundant in non-NE SCLC compared to NE SCLC in both datasets (Fig. 1A). Next, we performed RNA sequencing on CAFs educated by NE SCLC cell line H69 and non-NE SCLC cell line H196 in vitro. The results showed that MRC5 cells co-cultured with H196 (MRC5_coH196) had significantly more DEGs compared to those co-cultured with H69 (MRC5_coH69) (4011 vs. 42) (Fig. 1B). Additionally, the DEGs between MRC5_coH196 and MRC5_coH69 were distinct (Fig. 1C). PCA analysis confirmed significant differences in the expression profiles between MRC5_coH196 and MRC-5 cells, whereas MRC5_coH69 and MRC-5 cells showed similar transcriptomes (Fig. 1D). This trend was further validated by the heatmap analysis (Fig. 1E). These findings indicate that CAFs in NE and non-NE SCLC are heterogeneous in both abundance and transcriptome. The greater abundance and number of DEGs in non-NE SCLC-derived CAFs suggest more frequent and crucial crosstalk between CAFs and non-NE SCLC cells compared to NE SCLC cells.

### Crosstalk between CAFs and non-NE SCLC cells promotes glycolysis via FGF/FGFR signaling

**Fig. 2:**
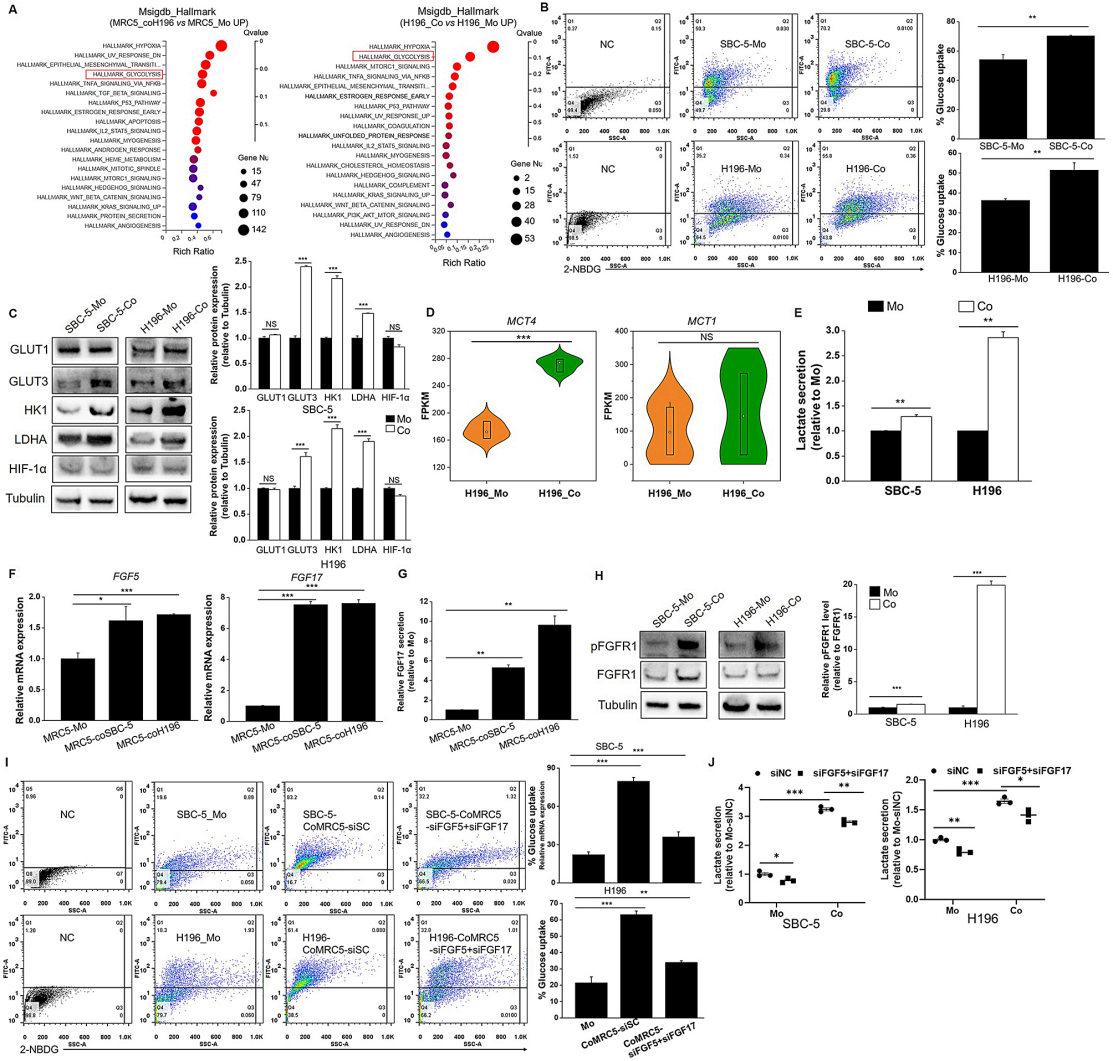
Glucose metabolic reprogramming during crosstalk between CAFs and non-NE SCLC cells. (**A**) MSigDB_Hallmark enrichment analysis was performed in upregulated DEGs of MRC5coH196 vs. MRC5_Mo cells and upregulated DEGs of H196_Co vs. H196_Mo cells. (**B**) SBC-5 and H196 cells co-cultured with MRC5 cells or individually cultured for 5 days, the uptake of 2-NBDG in SBC-5 and H196 cells was detected by flow cytometry. (**C**) The expression of glycolytic proteins in co-cultured and individually cultured SBC-5 and H196 cells was determined using western blot analysis, quantitative analyses of blots were performed based on triple repeated experiments. (**D**) The FPKM expression level of MCT4 and MCT1 in H196_Mo and H196_Co cells. (**E**) Lactate concentration in culture media of individually cultured (MRC5 + H196 or SBC-5) or co-cultured (SBC-5-CoMRC5 or H196-CoMRC5) cells was determined and normalized to the Mo group. (**F**) Expression of FGF5 and FGF17 in co-cultured and individually cultured MRC5 cells was determined by qRT-PCR. (**G**) FGF17 level in culture media of co-cultured and individually cultured MRC5 cells was determined by ELISA. (**H**) The phosphorylated FGFR1 and expression of FGFR1 in co-cultured and individually cultured SBC-5 and H196 cells was determined using western blot analysis. Quantitative analyses of blots were performed based on triple repeated experiments. (**I**) The uptake of 2-NBDG in SBC-5 and H196 cells which co-cultured with MRC5 cells transfecting with siRNAs for FGF5 and FGF17 or scramble controls, and individually cultured SBC-5 and H196 cells was determined. (**J**) MRC5 cells transfected with siRNAs for FGF5 and FGF17, then co-cultured or individually cultured with SBC-5 or H196 cells. Lactate concentration in culture media of individually cultured or co-cultured cells was determined and normalized to the Mo-scramble group. *P < 0.05, **P < 0.01, ***P < 0.001

To take an insight into the biological effect of the crosstalk between non-NE SCLC cells and CAFs, the cancer hallmark signaling enrichment analysis was performed. Both MRC5_coH196 and H196_Co cells showed enrichment in glycolysis-related genes (Fig. 2A), heatmaps showed the significant upregulation of these genes in MRC5_coH196 compared to MRC5_Mo cells and in H196_Co cells compared to H196-Mo cells (Fig S1, Supplementary Table S2). Increased glucose uptake and upregulation of key glycolytic pathway molecules in H196_Co and SBC-5_Co cells further indicated that CAFs promote glycolysis in non-NE SCLC cells (Fig. 2B-C). To further confirm this result, we knocked down of HK1, the key enzyme in glycolysis, in SBC-5 and H196 cells (Fig S2A), and found the increased glucose uptake in co-cultured SBC-5 and H196 cells was inhibited by HK1 knockdown (Fig S2B), suggesting that CAFs promote the uptake of glucose by SBC-5 and H196 cells and metabolize it through glycolysis. Metabolic symbiosis between glycolytic CAFs and tumor cells is known to promote oxidative phosphorylation (OXPHOS) in tumor cells through exploiting CAF-derived lactate(Li et al. 2021). However, our study found that H196_Co cells significantly upregulated *MCT4* (lactate exporter), but not *MCT1* (lactate importer), compared to H196_Mo cells (Fig. 2D). Moreover, the higher expression of *MCT4* over *MCT1* in both MRC5_coH196 and H196_Co cells (Fig. S2C) suggests that lactate secreted by CAFs probably accumulates in the culture medium rather than being utilized by SCLC cells. The significantly increased lactate concentration in co-culture system further confirmed the extracellular lactate accumulation (Fig. 2E). Moreover, the increased lactate concentration in co-culture media was reversed by HK1 knockdown in SBC-5 or H196 cells (Fig S2D), indicating that CAF promote glycolysis in non-NE SCLC cells and thereby induce lactate accumulation in extracellular environment.

As MRC5_coH196 and H196_Co cells were enriched in hypoxia pathway genes (Fig. 2A), we were wondering if excessive lactate secretion might induce a pseudo-hypoxic condition, stabilizing hypoxia-inducible factor HIF-1α and promoting glycolysis. However, no changes in HIF-1α protein levels were observed in both H196 and SBC-5 cells after co-culture with MRC5 cells (Fig. 2C). Instead, the increased expression and secretion of FGF5 and FGF17 in MRC5_coH196 and MRC5_coSBC-5 cells caught our attention (Fig. 2F-G, Fig S3A). Recent study has shown that FGF/FGFR signaling is a dominant interaction in the non-NE TME of SCLC(Desai et al. 2024). For this, we evaluate if FGF from CAFs was involved in the glycolysis of SCLC cells. Phosphorylation of FGFR1, the receptor for FGF5 and FGF17, was increased in both H196-Co and SBC-5-Co cells compared to H196-Mo and SBC-5-Mo cells (Fig. 2H), indicating that FGF from CAFs indeed activates FGFR1 pathway in non-NE SCLC cells. Moreover, knockdown of FGF5 and FGF17 in MRC5 cells by siRNA reversed the promotion of glucose uptake in SBC-5 and H196 cells by MRC5 (Fig S3B, Fig. 2I), the increase of lactate concentration in media of co-cultured MRC5 cells was also attenuated after FGF5 and FGF17 knockdown in MRC5 cells (Fig. 2J). Furthermore, treatment with anlotinib, a tyrosine kinase inhibitor targeting FGFR1, significantly inhibited FGFR1 phosphorylation in H196 and SBC-5 cells upon co-culture with MRC5 cells (Fig S3C), reducing the expression of LDHA (Fig S3C). The uptake of 2-NBDG was also inhibited by anlotinib in both H196 and SBC-5 cells upon co-culture with MRC5 cells (Fig S3D. These findings suggest that CAFs promote glycolysis in non-NE SCLC cells via FGF secretion, activating the FGFR1 pathway.

Previous studies have shown that many cancer cells modulate the metabolic phenotype of CAFs from OXPHOS to glycolysis by altering the expression of CAV1, IDH3A, or ITGB4(Bonuccelli et al. 2010; Sung et al. 2019; Zhang et al. 2015). Interestingly, our data showed a significant decrease in CAV1 and IDH3A expression and a slight increase in ITGB4 expression in MRC5_coH196 cells compared to MRC5_Mo cells (Fig. S4), suggesting similar mechanisms in SCLC.

### CAFs promote glycolysis-mediated STING signaling activation in non-NE SCLC cells

**Fig. 3:**
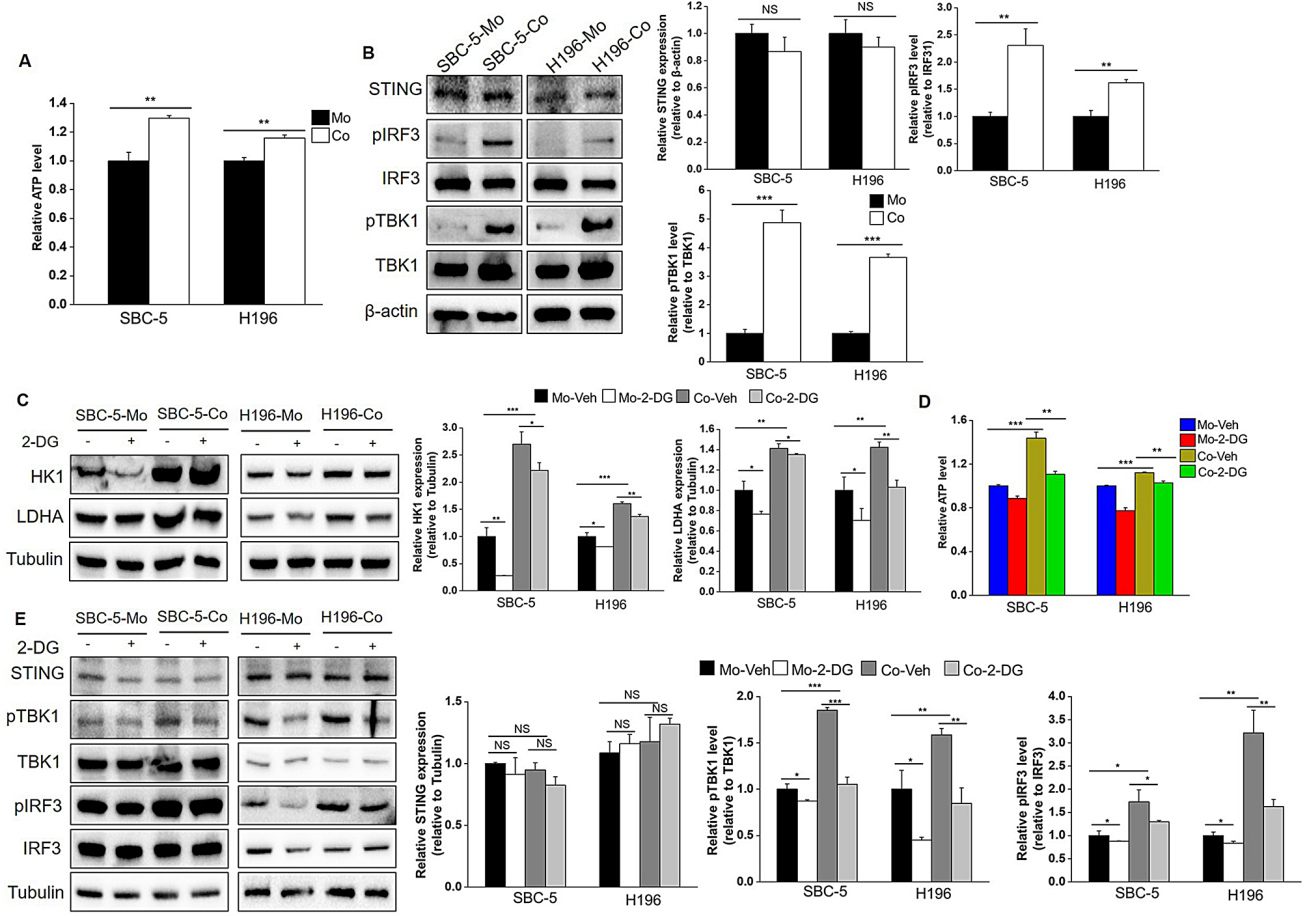
Glycolysis mediates STING signaling activation during crosstalk between CAFs and non-NE SCLC cells. (**A**) ATP production in co-cultured and individually cultured SBC-5 and H196 cells was detected using ATP Luminescent reagent. (**B**) Total and phosphorylated proteins of STING signaling was determined by western blot analysis. (**C**-**E**) Co-cultured and individually cultured SBC-5 and H196 cells were treated with 2-DG, the expression of glycolytic proteins (**C**), ATP production (**D**) and STING signaling in SBC-5 and H196 cells were determined. *P < 0.05, **P < 0.01, ***P < 0.001

We observed a significant increase in ATP levels in SBC-5-Co and H196-Co cells (Fig. 3A), likely due to enhanced glycolysis. ATP is the crucial precursor of 2′,3′-cyclic GMP-AMP (cGAMP), which activates the STING signaling pathway. We confirmed this by noting elevated phosphorylation levels of TBK1 and IRF3 in SBC-5-Co and H196-Co cells (Fig. 3B), indicating STING signaling activation. To further validate the role of glycolysis in this process, we treated SBC-5 and H196 cells with 2-DG to inhibit glycolysis during both co-culture and individual culture conditions. The expression of key enzymes of glycolysis, HK1 and LDHA, significantly reduced in both cell lines after 2-DG treatment (Fig. 3C). Moreover, the increased ATP levels in SBC-5-Co and H196-Co cells were reduced following 2-DG treatment (Fig. 3D). As expected, the phosphorylation levels of TBK1 and IRF3 were also attenuated in both cell lines after 2-DG treatment (Fig. 3E). These findings indicate that CAF-induced glycolytic ATP production promotes the STING signaling pathway in non-NE SCLC cells.

### CAF-induced STING signaling elicits T cell chemo-attractant expression but does not affect CD8 + T cell numbers

**Fig. 4:**
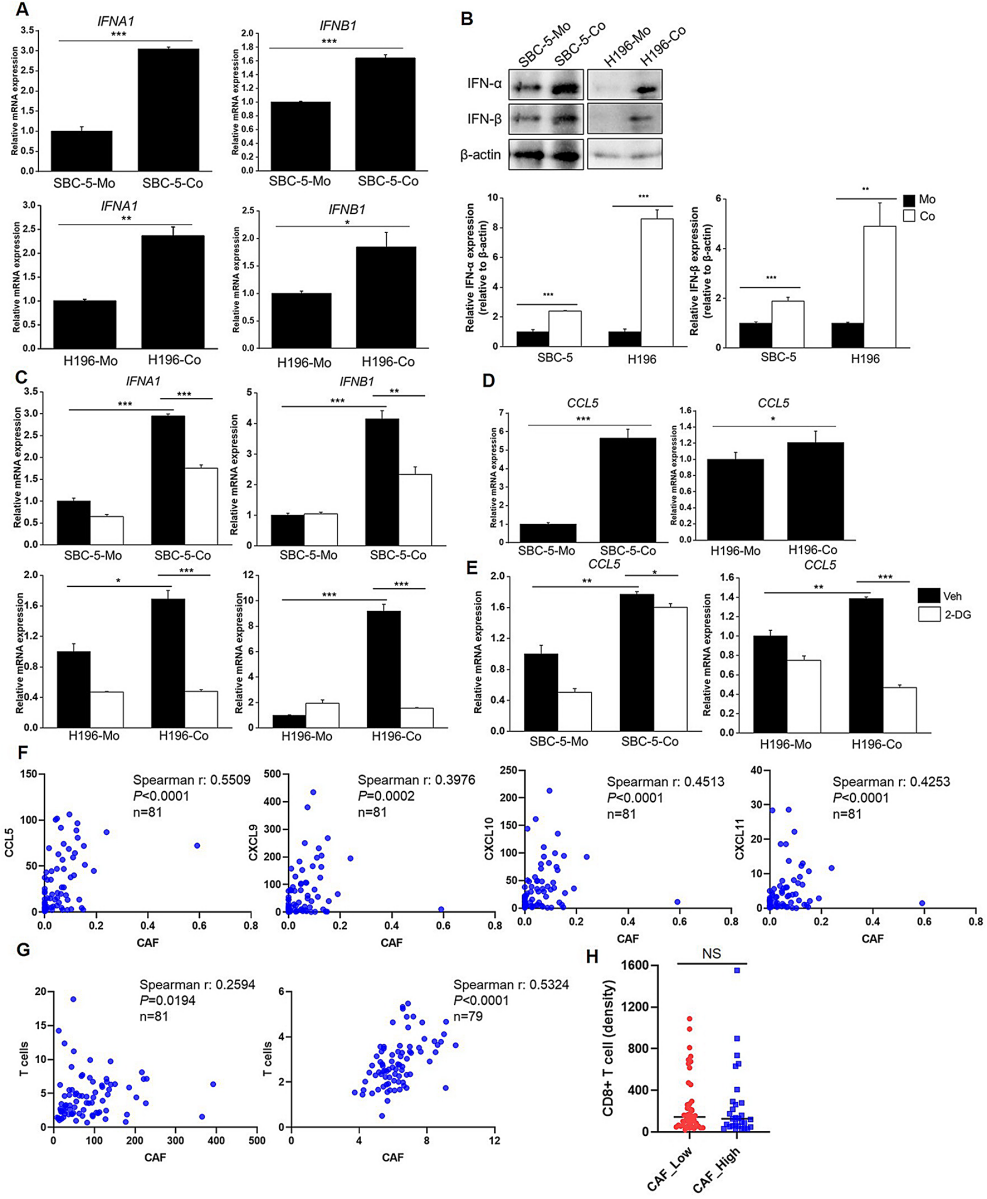
CAFs promoted T cell chemo-attractants expression. (**A**, **D**) Expression of IFN I (**A**) and CCL5 (**D**) in co-cultured and individually cultured SBC-5 and H196 cells was determined by qRT-PCR. (**B**) The protein levels of IFN-α and IFN-β in co-cultured and individually cultured SBC-5 and H196 cells were determined by western blot. (**C**, **E**) After treatment with 2-DG, expression of IFN I (**C**) and CCL5 (**E**) in co-cultured and individually cultured SBC-5 and H196 cells was measured using qRT-PCR. (**F**) Spearman correlation was performed to measure the correlation between expression of T cell chemo-attractants and CAF abundance in George’s cohort. (**G**) Correlation between T cell abundance and CAF abundance estimated by MCP-counter was analyzed using Spearman correlation in George’s cohort and GSE60052 dataset. (**H**) SCLC tissue samples in tissue array (*n* = 74) were divided into CAF-High and CAF-Low groups based on the median value of α-SMA IHC score, the density of CD8 + T cells was compared between CAF-High and CAF-Low groups. *P < 0.05, **P < 0.01, ***P < 0.001

STING signaling is known to activate type I interferons (IFNs). Actually, the enhancement of *IFNA1* and *IFNB1* mRNA and protein expression was observed in both SBC-5-Co and H196-Co cells (Fig. 4A-B). Moreover, treatment with 2-DG attenuated this increasement (Fig. 4C), indicating a link between glycolysis and IFN expression. Additionally, the T cell chemo-attractant CCL5, a target of STING signaling, was significantly upregulated in SBC-5 and H196 cells after co-culture with MRC5 cells (Fig. 4D). Furthermore, Inhibition of glycolysis via 2-DG reduced CCL5 expression in both cell lines upon co-culture (Fig. 4E), suggesting that CAF-induced glycolysis may influence T cell infiltration. Further analysis showed a positive correlation between CAF abundance and the expression of T cell chemo-attractants, including CCL5, CXCL9, CXCL10, and CXCL11, in SCLC tissues from both George’s study and the GSE60052 dataset (Fig. 4F, Fig. S5). CAF abundance also positively correlated with T cell abundance in these datasets (Fig. 4G). However, IHC staining of CAF marker α-SMA and CD8 in SCLC tissue arrays revealed no significant difference in CD8 + T cell density between CAF-High and CAF-Low groups (Fig. 4H), indicating that CAF-activated STING signaling does not correlate with cytotoxicity T cell numbers.

### Antigen presentation features of CAFs educated by non-NE SCLC cells

**Fig. 5:**
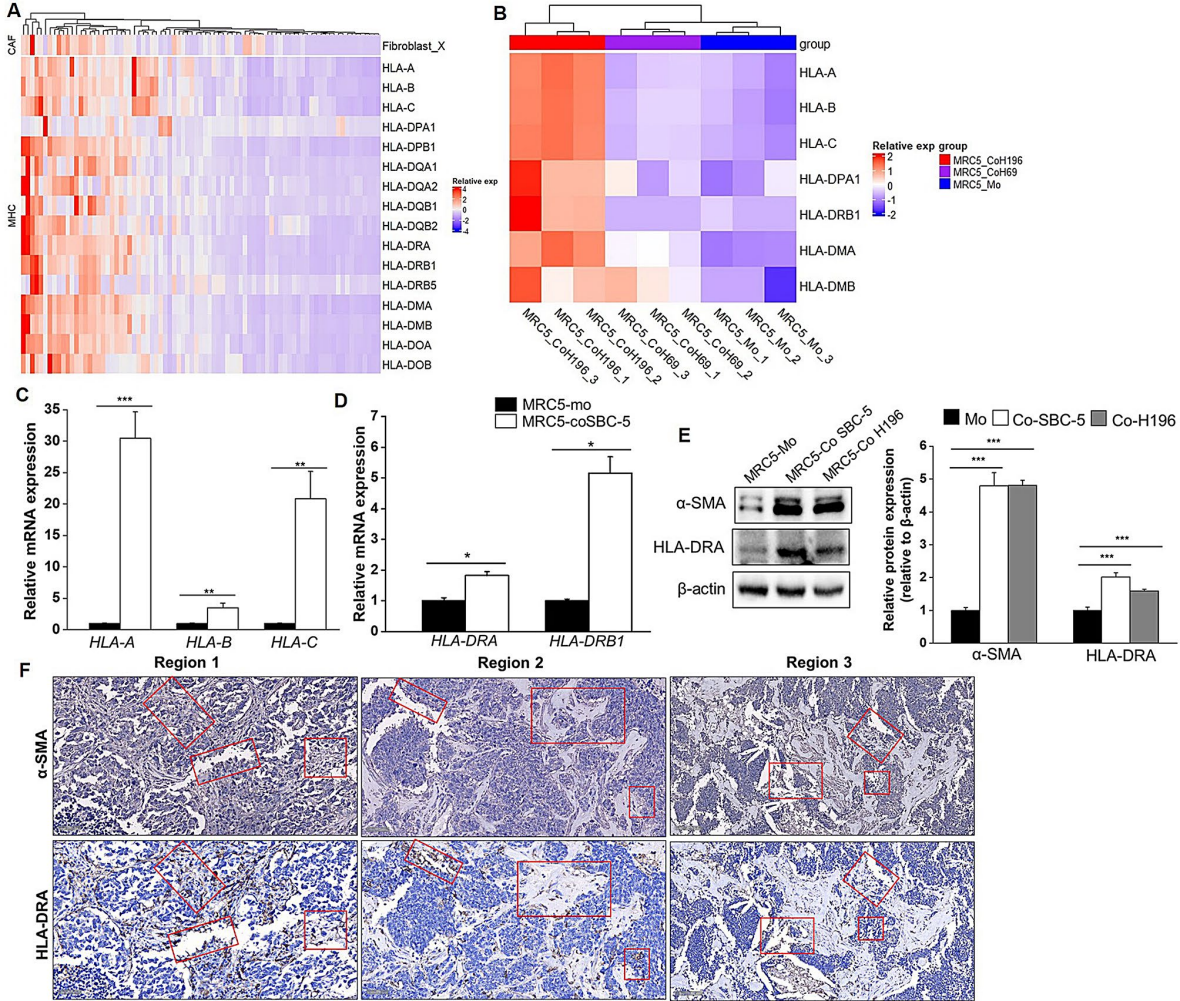
The apCAF exists in SCLC. (**A**) Heatmap presents the expression of HLA genes and fibroblast abundance in George’s cohort. (**B**) Differences in the expression of HLA-genes among MRC5_Mo cells, MRC5_coH69 cells and MRC5_coH196 cells. (**C**-**D**) The expression of HLA genes in MRC5_coSBC-5 cells and MRC5_Mo cells was determined by qRT-CPR. (**E**) The expression of α-SMA and HLA-DRA proteins in co-cultured and individually cultured MRC5 cells was detected by western blot. (**F**) CAF marker α-SMA and HLA-DRA were stained in SCLC tissue arrays by IHC, the regions where positive signals for α-SMA and HLA-DRA presented in the identical areas of consecutive slices were marked with red boxes. Scale bar: 50 μm. *P < 0.05, **P < 0.01, ***P < 0.001

Analyzing the transcriptome of SCLC tissues from George’s study, we found that cases with high CAF abundance expressed numerous antigen presentation genes (Fig. 5A). To determine whether these genes were expressed by CAFs, we examined the expression profile of CAF educated in vitro. Our findings revealed that many human leucocyte antigen (HLA) genes were expressed in MRC5_coH196 cells, but not in MRC5_Mo cells, and were scarce in MRC5_coH69 cells (Fig. 5B). Further analysis confirmed the upregulation of HLA-A, HLA-B, HLA-C, and HLA-DR mRNA in another non-NE SCLC cell line, SBC-5, after co-culture with MRC5 cells (Fig. 5C-D). Protein expression of HLA-DRA was also increased in both H196 and SBC-5 cells upon co-culture with MRC5 cells (Fig. 5E). As reported that an apCAF subpopulation expressing MHC molecules has been identified in various cancers, but not in SCLC. To validate the presence of this CAF subpopulation in SCLC, we performed IHC staining of α-SMA and HLA-DRA in consecutive SCLC tissue sections, which with non-NE characteristics according to our previous studies(Lu et al. 2024), showing that REST, the non-NE marker, was expressed in all these samples, while CD56, the NE marker, was only weak expression (Fig S6A). As expected, some identical regions in consecutive sections were positively stained for both α-SMA and HLA-DRA (Fig. 5F), suggesting the presence of apCAFs in SCLC.

### ApCAFs and T cell anergy in SCLC

**Fig. 6:**
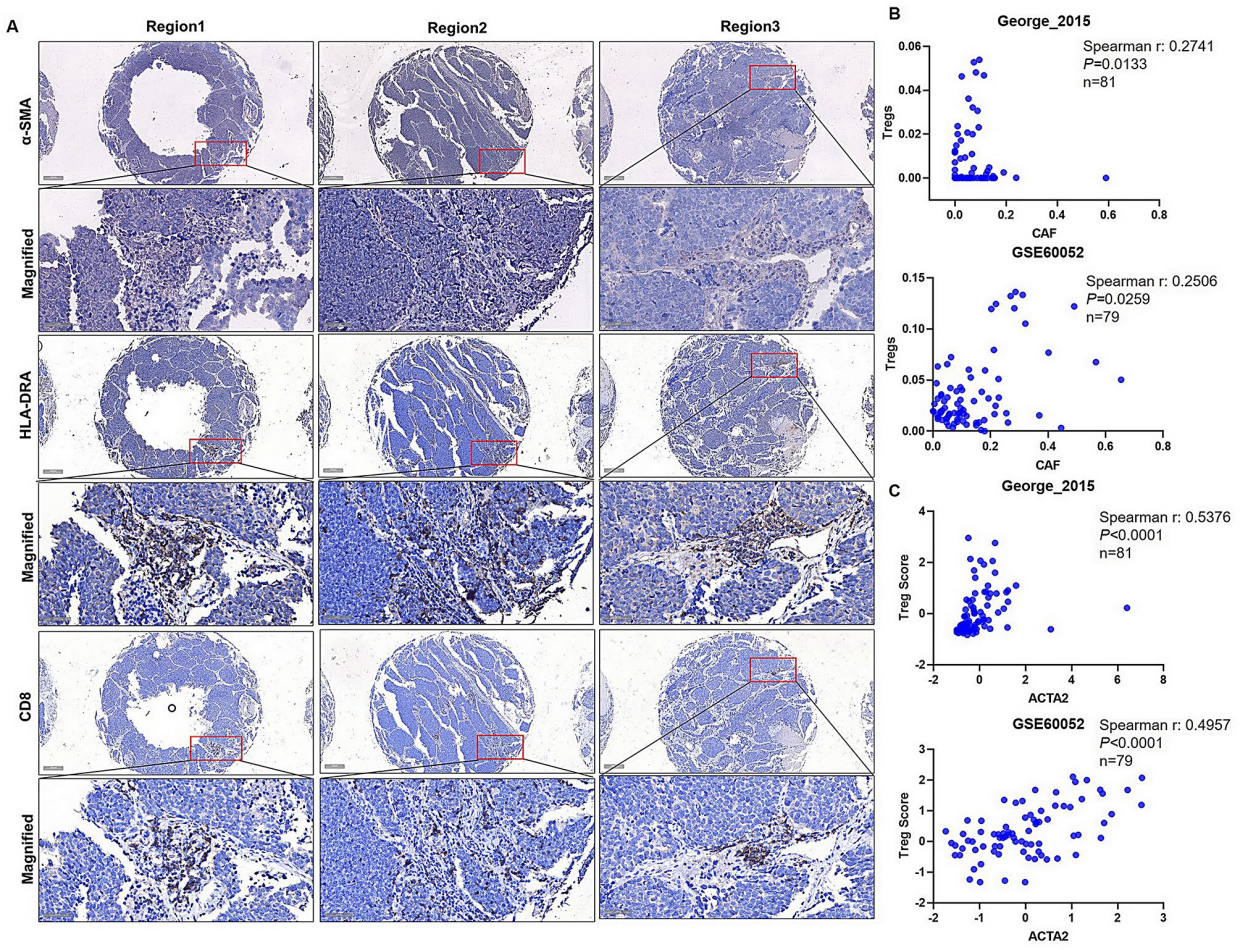
CAFs involved in trapping CD8 + cells and Treg differentiation. (**A**) α-SMA, HLA-DRA and CD8 were detected in SCLC tissue arrays using IHC, the regions with presence of positive signals for α-SMA, HLA-DRA and CD8 were marked with red boxes. Scale bar: 200 μm and 50 μm (Magnified). (**B**) Correlation between CAF abundance and Treg abundance estimated by xCell algorithm from George’s cohort and GSE60052 dataset was performed using Spearman correlation. (**C**) Correlation between the expression of ACTA2 and Treg score, which calculated as the geometric mean of CD25 and FOXP3 expression, was analyzed by Spearman correlation

Fibroblasts are non-professional antigen presenting cells (APC) that are capable of presenting antigen to T cells under specific condition, apCAFs in various cancers have demonstrated this capability(Elyada et al. 2019a, b; Harryvan et al. 2022a, b). Our findings suggest the presence of apCAF subpopulations in SCLC. However, neither quiescent MRC5 cells nor co-cultured MRC5 cells expressed CD80 and CD86, the co-stimulatory molecules essential for T cell activation, consistent with previous reports(Elyada et al. 2019a, b; Harryvan et al. 2022a, b). This suggests that apCAFs might capture T cells but render them anergic. To evaluate if apCAFs trap T cells in SCLC, we performed IHC staining of α-SMA, HLA-DRA, and CD8 on three consecutive SCLC tissue sections, which with non-NE characteristics according to our previous studies (Fig S6B) (Lu et al. 2024). Notably, some identical stroma regions in the three slices presented positive staining for these three proteins (Fig. 6A), suggesting that apCAFs in SCLC likely trap cytotoxic T cells in stroma, potentially preventing them from killing tumor cells. Additionally, APCs lacking co-stimulatory molecules can induce naïve CD4 + T cells to become Tregs in an antigen-specific manner (Huang et al. 2022). To explore this possibility, we examined the correlation between CAF abundance and Treg abundance in SCLC tissues from George’s study and the GSE60052 datasets. There was a significant positive correlation between CAF abundance and Treg abundance (Fig. 6B). Furthermore, the expression of *ACTA2*, the gene encoding α-SMA, was also positively correlated to Treg score, based on the expression of *CD25* and *FOXP3*, the Treg markers (Fig. 6C). These data indicate that CAFs in SCLC might promote the production of Tregs, warranting further research into whether this occurs in an antigen-specific manner.

## Discussion

Recent research by Parth and colleagues highlighted the crucial role of CAFs in contributing to TME heterogeneity and immune exclusion in SCLC using spatial transcriptomics(Desai et al. 2024). However, the mechanisms by which CAFs drive SCLC progression remain poorly understood, mostly due to the challenge of obtaining sufficient tumor tissue for in-depth research. Noteworthy, fibroblasts educated by cancer cells in vitro have been effectively used as CAF models in multiple studies(Ferraresi et al. 2024; Harryvan et al. 2022a, b; Öhlund et al. 2017). In this study, we integrated in vitro-educated CAFs, publicly available datasets, and SCLC tissue arrays to elucidate the heterogeneity in abundance and transcriptome profiles of CAFs derived from NE and non-NE SCLC, revealed the underlying mechanisms during crosstalk between CAFs and non-NE SCLC cells or T cells.

Accumulating evidence suggests that CAFs are major regulators of tumor metabolism reprogramming, particularly glucose metabolism(Chiarugi et al. 2016). The metabolism coupling between CAFs and tumor cells can alter tumor cell behavior and shape the immune microenvironment. CAF activation often involves a switch to glycolysis, with the modulation of CAV1, IDH3A, and ITGB4 expression and the activation of TGF-β signaling(Bonuccelli et al. 2010; Guido et al. 2012; Sung et al. 2019; Zhang et al. 2015). In our findings, the increased α-SMA expression in MRC5 cells upon co-culture with H196 cells suggests that MRC5 cells are activated to CAFs (Fig. 6E), co-cultured MRC5 cells indeed enriched multiple glycolysis genes. The downregulation of CAV1 and IDH3A and upregulation of ITGB4 in MRC5_coH196 cells compared to MRC5_Mo cells indicate that SCLC cells might regulate CAF glucose metabolism primarily through these genes, while the TGF-β signaling was not enriched in MRC5_coH196 cells. These findings are consistent with Parth’s study that that CAF subpopulation in hybrid-NE SCLC showed a glycolytic signature(Desai et al. 2024). Moreover, our data showed that glycolysis in non-NE SCLC cells is promoted by CAFs via FGF/FGFR1 signaling, not by HIF-1α, a key regulator of hypoxia-induced glycolysis. The role of FGF/FGFR signaling in promoting glycolysis has been reported in other cancers (Ye et al. 2024; Yu et al. 2017), whereas our finding emphasize that CAF-derived FGFs can promote glycolysis in SCLC cells via FGFR1 signaling.

Previous studies have highlighted the metabolic couplings between tumor cells and CAFs, suggesting that tumor cells generally take up metabolites secreted by CAFs like pyruvate and lactate as alternative carbon source to take place glucose(Fiaschi et al. 2012). In this condition, the importer of lactate, MCT1, should be upregulated in tumor cells, and OXPHOS should be the dominant glucose metabolic phenotype in tumor cells (Shi et al. 2015). However, we found no significant change in MCT1 expression in H196 cells after co-culture with MRC5 cells. Instead, the promotion of glycolysis and upregulation of lactate exporter MCT4 were observed in non-NE SCLC cells, lactate accumulation in co-culture system media was also detected, indicating that non-NE SCLC cells did not exploit CAF-secreted lactate for respiration, but uptake glucose to glycolysis and secrete lactate into TME. Notably, substantial evidence suggests that an acid TME result from lactate is detrimental to normal cells, including immune cells(Wang et al. 2020). Moreover, the elicited glycolysis in tumor cells aggravates the competition for glucose between tumor cells and immune cells, further impair cytotoxicity of immune cells to tumor cells(Espelage et al. 2024). Despite the immunosuppressive potential of the lactate-rich TME, glycolysis-derived ATP in non-NE SCLC cells activated STING signaling, increasing IFN-I and chemo-attractant expression. Although the CAF abundance had no relationship with CD8 + T cell counts in SCLC tissues, indicating the presence of other mechanisms to impact T cell function(Zhang et al. 2011), these findings indicate the complex role of CAF in SCLC immunity.

Interestingly, our data elucidate the presence of apCAF in SCLC, non-professional APCs capable of presenting antigens to CD8 + and CD4 + T cells without co-stimulatory molecules. Due to their inability to activate T cells, apCAFs may catch CD8 + T cells and trap them in stroma area, or induce naïve CD4 + T cells to Tregs, in an antigen-specific manner(Harryvan et al. 2022a, b; Huang et al. 2022). Notably, in our SCLC tissue arrays, many CD8 + T cells co-located with apCAFs in the stroma, and CAFs were positive correlated with Tregs in SCLC cases from George’s study and GSE60052 dataset. These findings indicate that SCLC-derived apCAFs may make T cell anergy. Combining with the previous findings, CAFs in SCLC may promote T cell infiltration by increasing the expression of chemo-attractants, but apCAFs may trap them in stroma or induce Treg production. Moreover, the excessive lactate in TME caused by glycolysis may impair the cytotoxic function of T cells toward tumor cells.

This study has several limitations. Firstly, though we used cell lines, tissue arrays and publicly available datasets to explore the CAF function in SCLC, cell lines, especially commercially sourced fibroblasts, cannot totally reflect the true characteristics of the cells in vivo. Primary CAFs from SCLC patient tissues are needed to validate our findings. However, tissues from SCLC patients are difficult to access. Secondly, animal models should be used to explore the antigen-presenting capability of CAFs and their effects on T cell behavior. Further research using more precise experimental methods is necessary to validate our speculations.

## Conclusion

**Fig. 7:**
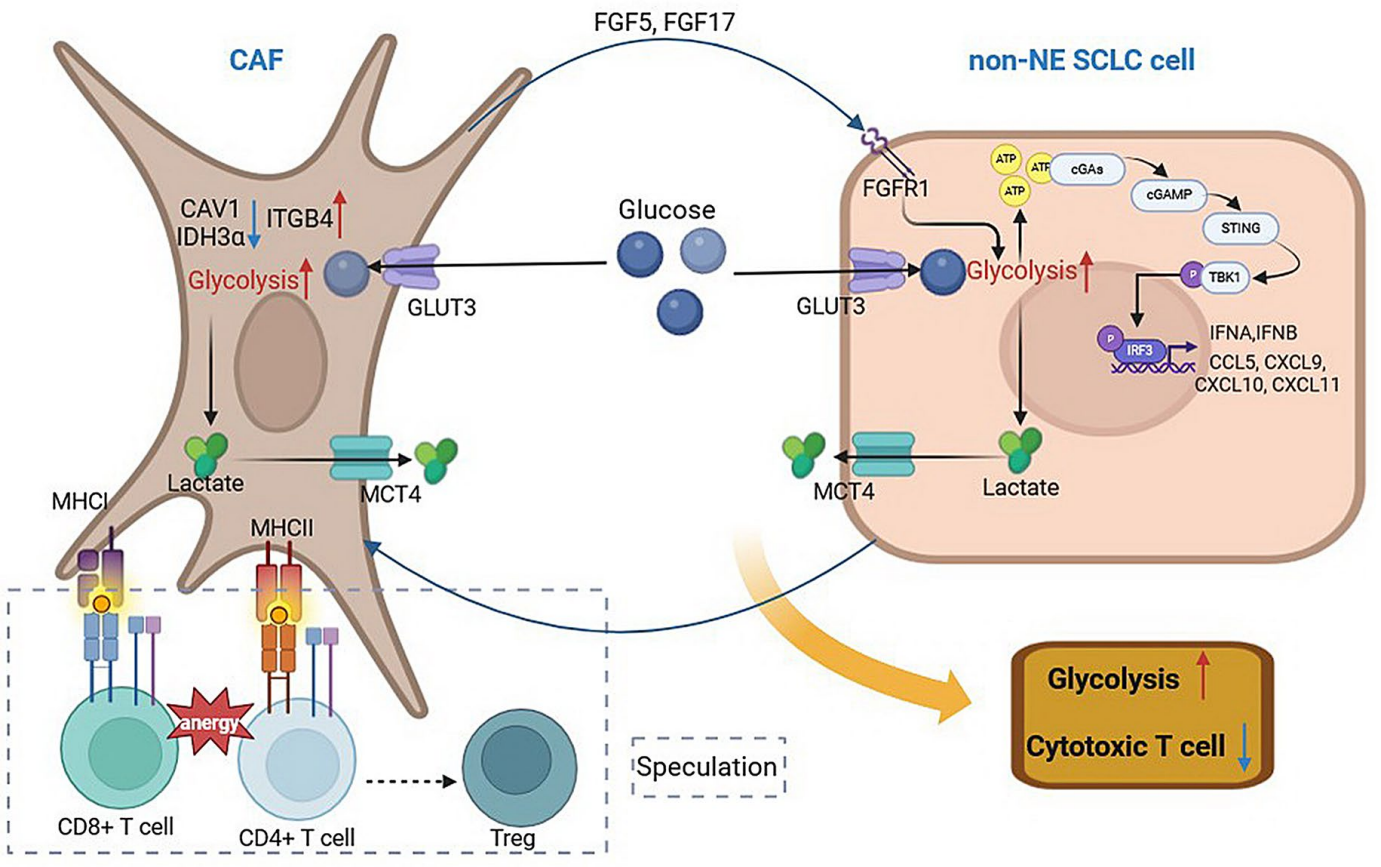
Schematic model on the crosstalk between CAF and non-NE SCLC cell and its’ impact on glycolysis and T cell function

This study provides crucial insights into the crosstalk between CAFs and SCLC cells, an area that is poorly understood. Our findings demonstrate that interaction between CAFs and tumor cells is more likely in non-NE SCLC, triggering metabolic reprogramming. CAFs promote glycolysis in non-NE SCLC cells via FGF/FGFR signaling, activating STING signaling and increasing T cell chemo-attractant expression, potentially facilitating T cell infiltration but not CD8 + T cell infiltration. Additionally, we found apCAFs in SCLC tissues co-localized with some CD8 + T cells, suggesting that CAFs might trap CD8 + T cells in the stroma or induce them to become Tregs, impairing their cytotoxic function (Fig. 7). This study extends the understanding to the pro-tumor role of CAF in SCLC, which may reveal the potential therapeutic targets to improve treatment response of SCLC patients.

## Electronic supplementary material

Below is the link to the electronic supplementary material.

Supplementary Material 1

Supplementary Material 2

## Data Availability

Sequence data that support the findings of this study have been deposited in the Gene Expression Omnibus with the primary accession code GSE274458.

## Abbreviations

APC: Antigen presenting cell
apCAF: Antigen-presenting cancer-associated fibroblast
CAF: Cancer-associated fibroblast
cGAMP: 2′,3′-cyclic GMP-AMP
DEG: Differentially expressed gene
EV: Extracellular vesicle
HLA: Human leucocyte antigen
ICI: Immune checkpoint inhibitor
IFN: Type I interferon
IHC: Immunohistochemical staining
MDSC: Myeloid-derived suppressor cell
MHC: Major histocompatibility complex
NE: Neuroendocrine
OXPHOS: Oxidative phosphorylation
RNA-seq: RNA sequencing
SCLC: Small Cell Lung Cancer
TME: Tumor microenvironment
Treg: Regulatory T cell
