# Supplementary material for: Crosstalk between cancer-associated fibroblasts and non-neuroendocrine tumor cells in small cell lung cancer involves in glycolysis and antigen-presenting features
Source: Mol Med. 2024 Dec 25;30:274. doi: 10.1186/s10020-024-01051-y (PMC11669202; doi:10.1186/s10020-024-01051-y)
Supplement: Supplementary file 2 — Supplementary Material 2 [file 10020_2024_1051_MOESM2_ESM.docx]

**Supplementary information**

Crosstalk between cancer-associated fibroblasts and non-neuroendocrine tumor cells in small cell lung cancer involves in glycolysis and antigen-presenting features

Yuanhua Lu ^1^, Hui Li ^2^, Peiyan Zhao ^2^, Xinyue Wang ^1^, Wenjun Shao ^1^, Yan Liu ^2^, Lin Tian ^1^, Rui Zhong ^2^, Haifeng Liu ^3*^, Ying Cheng ^2 4*^

**
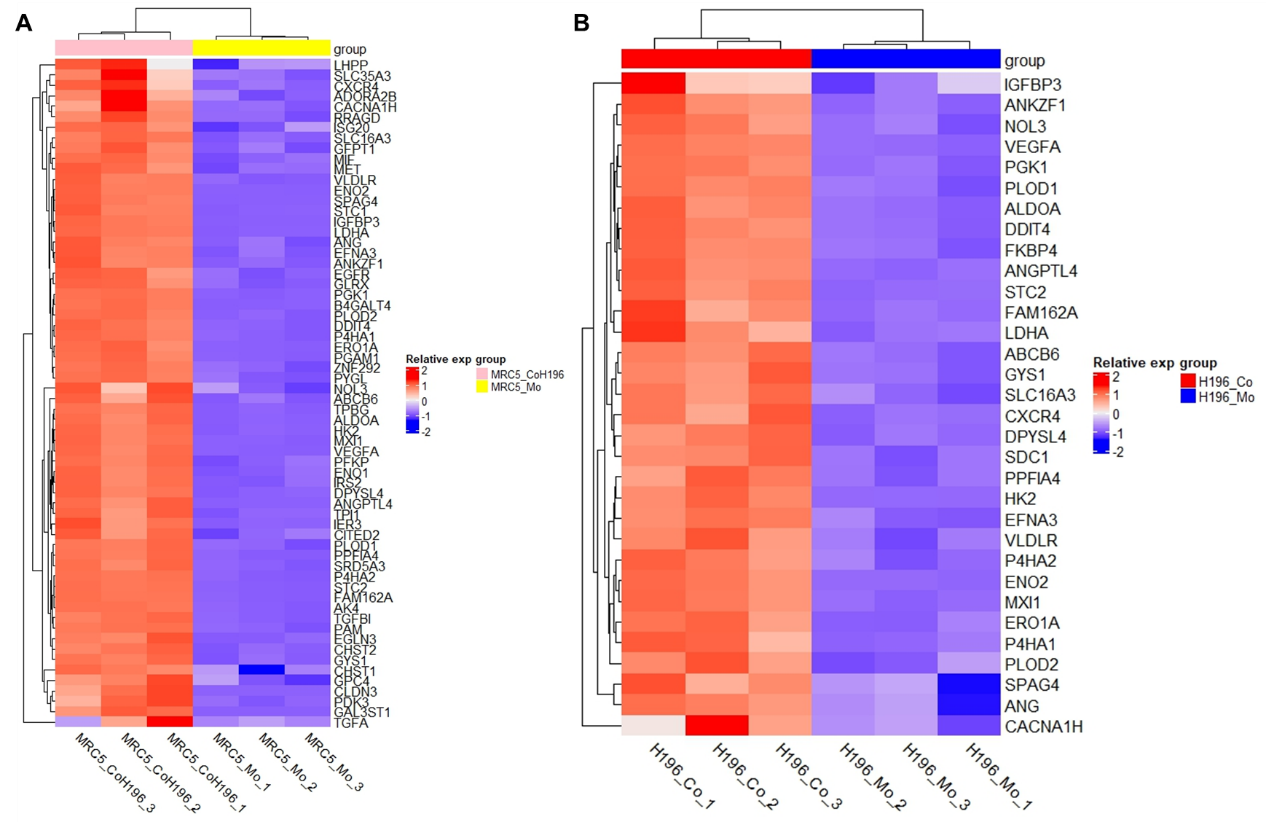
Figure S1**

Expression profile of glycolytic genes in co-cultured and individually cultured MRC5 and H196 cells. (A-B) Heatmap presents the expression of glycolytic genes in MRC5_Mo, MRC5_CoH196, H196-Mo, H196_Co cells.

**Fig S2**


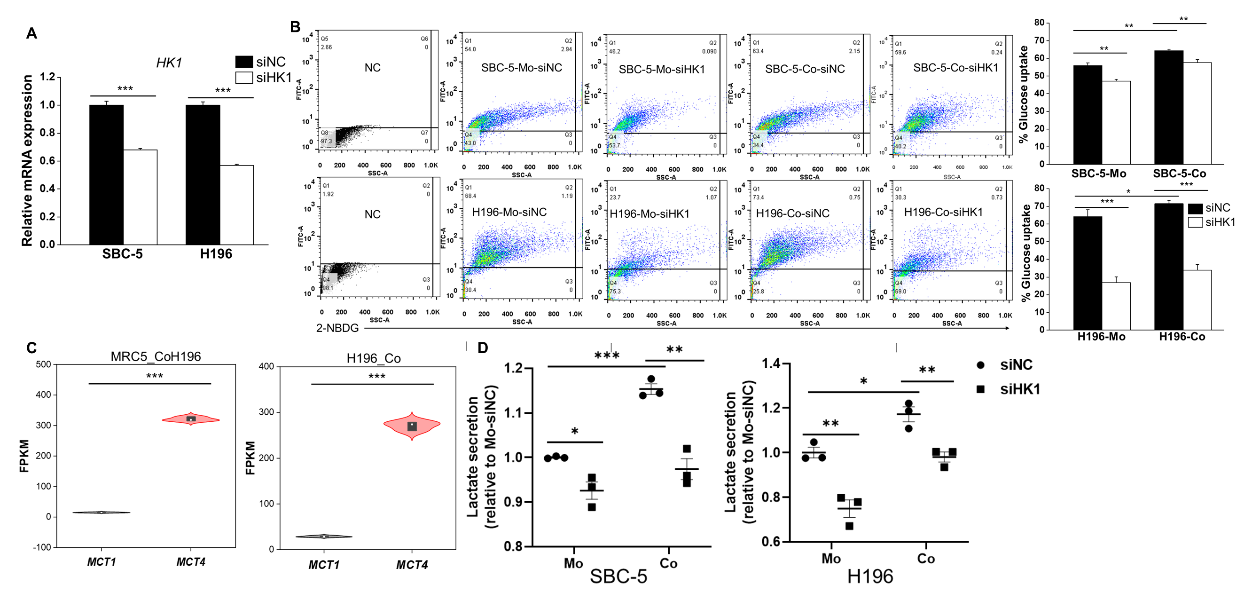
CAF promote glycolysis in non-NE SCLC cells. (A) The mRNA expression level of HK1 in SBC-5 and H196 cells after transfection with siRNA for HK1 was determined by qRT-PCR. (B) The uptake of 2-NBDG in co-cultured and individually cultured SBC-5 and H196 cells upon siHK1 or si-Scramble transfection. (C) The comparison between FPKM expression level of MCT1 and MCT4 in MCR5_coH196 and H196_Co cells. (D) Lactate concentration in co-cultured and individually cultured SBC-5 and H196 cell media upon siRNA for HK1 transfection. **P*<0.05, ***P*<0.01, ****P*<0.001.

**Figure S3**


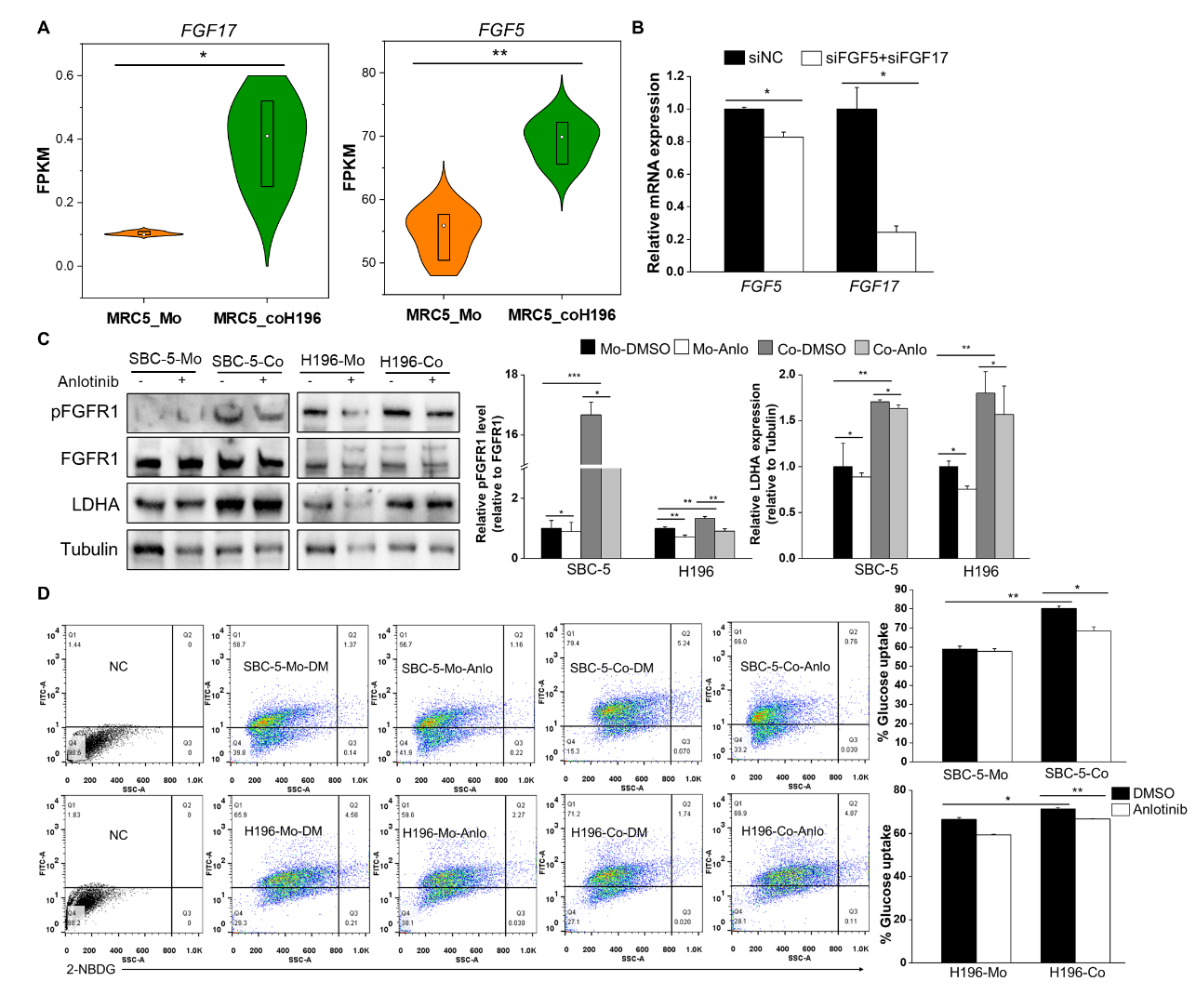
FGF/FGFR1 pathway mediates CAF promotion of glycolysis in non-NE SCLC cells. (A) The comparison between FPKM expression level of FGF5 and FGF17 in MCR5_Mo and MRC5_CoH196 cells. (B) The mRNA expression level of FGF5 and FGF17 in MRC5 cells after transfection with siRNA for FGF5 and FGF17 was determined by qRT-PCR. (C) The phosphorylated FGFR1 and expression of FGFR1 in co-cultured and individually cultured SBC-5 and H196 cells was determined using western blot analysis. (D) The uptake of 2-NBDG in co-cultured and individually cultured SBC-5 and H196 cells upon anlotinib treatment. **P*<0.05, ***P*<0.01, ****P*<0.001.

**Figure S4**


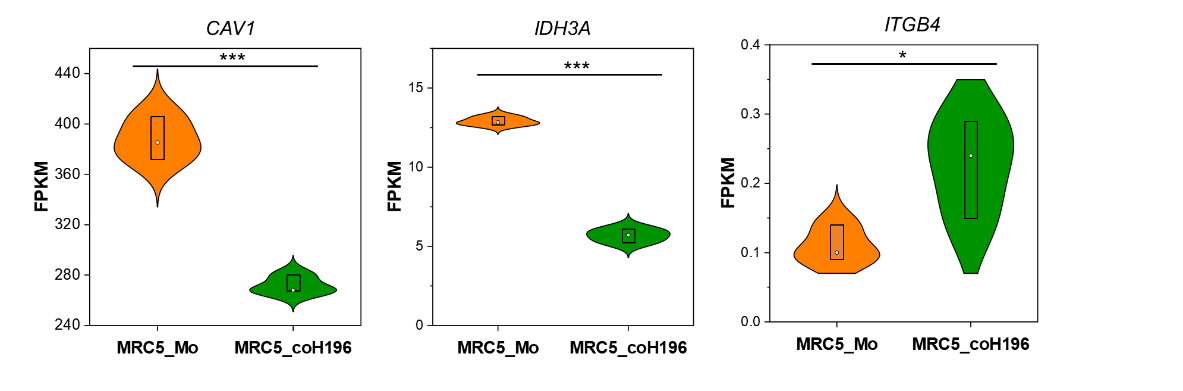
Expression of CAF metabolic phenotype regulatory factors. The comparison between FPKM expression level of *CAV1*, *IDH3A* and *ITGB4* in MCR5_Mo and MRC5_CoH196 cells. **P*<0.05, ***P*<0.01, ****P*<0.001.

**
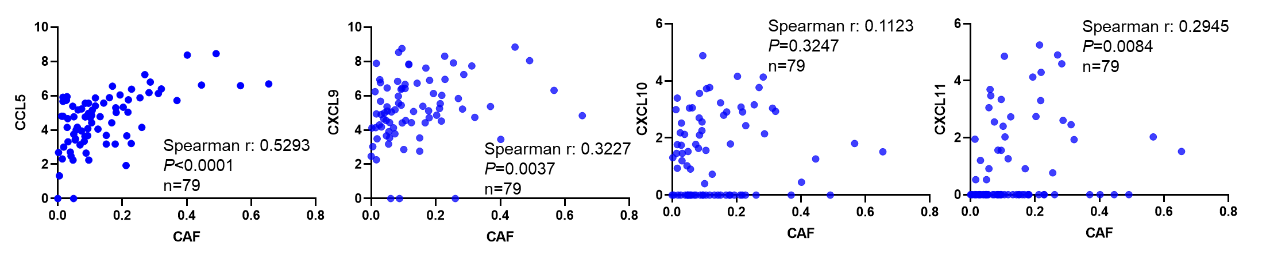
Figure S5**

The correlation between CAF abundance and expression of T cell chemo-attractants. Spearman correlation was performed to determine the correlation between CAF abundance and expression of CCL5, CXCL9, CXCL10, CXCL11 in GSe60052 dataset.

**
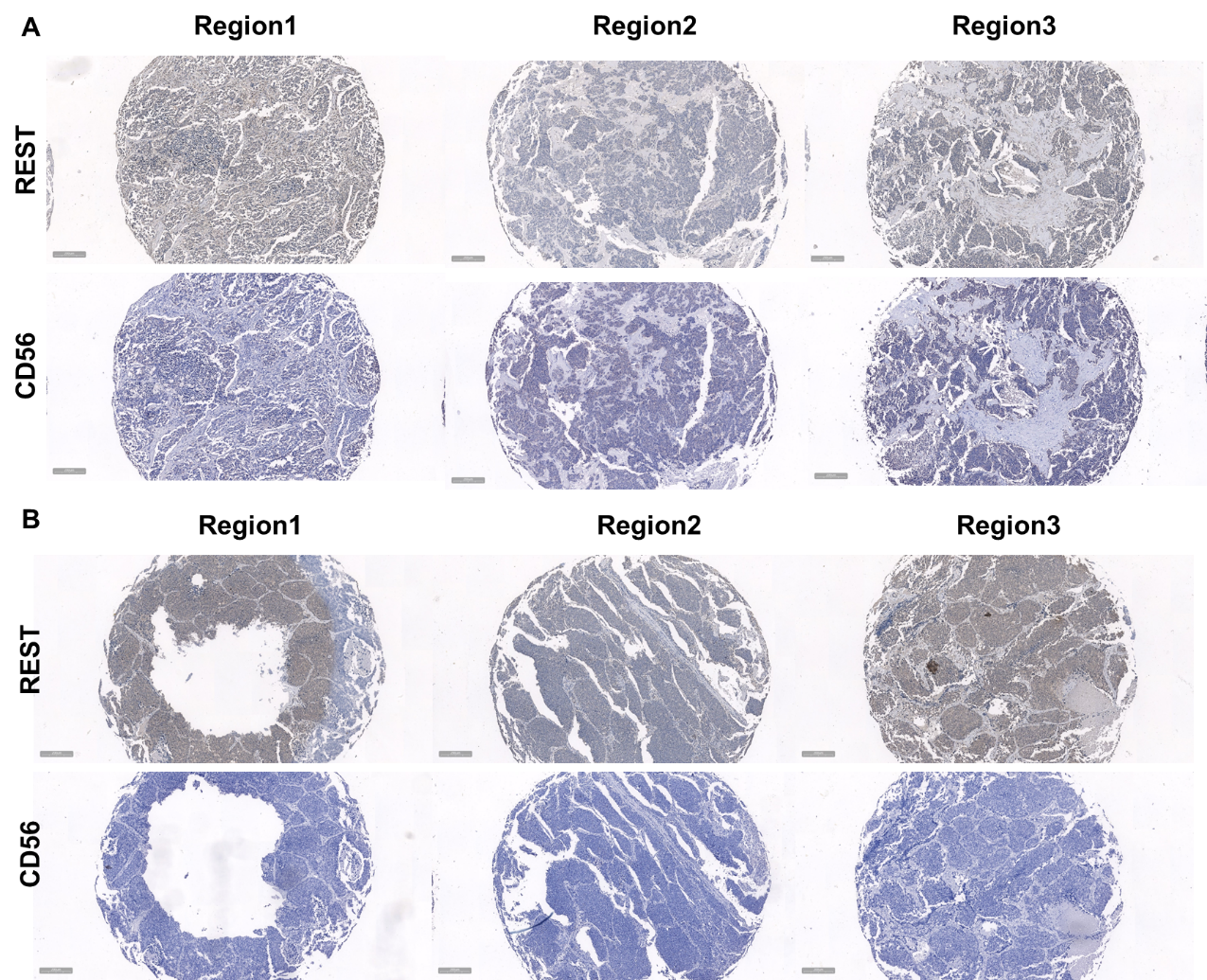
Figure S6**([Lu et al. 2024](#_ENREF_1))

Assessment of expression of NE and non-NE markers. (A-B) Expression of NE non-NE marker REST and NE marker CD56 in SCLC tissue arrays was detected by IHC.

**Premiers for qPCR**

| Genes | Sequences |
| --- | --- |
| *β-actin* | F: 5’- TCAGAAGGATTCCTATGTGGGCGA-3’ |
|  | R: 5’- TTTCTCCATGTCGTCCCAGTTGGT-3’ |
| *IFNA1* | F: 5’- GCCTCGCCCTTTGCTTTACT-3’ |
|  | R: 5’- CTGTGGGTCTCAGGGAGATCA-3’ |
| *IFNB1* | F: 5’- GCTTGGATTCCTACAAAGAAGCA-3’ |
|  | R: 5’- ATAGATGGTCAATGCGGCGTC-3’ |
| *CCL5* | F: 5’- CCAGCAGTCGTCTTTGTCAC-3’ |
|  | R: 5’- CTCTGGGTTGGCACACACTT-3’ |
| *HK1* | F: 5’-CACATGGAGTCCGAGGTTTATG-3’ |
|  | R:5’-CGTGAATCCCACAGGTAACTTC-3’ |
| *FGF5* | F:5’-CACTGATAGGAACCCTAGAGGC-3’ |
|  | R:5’-CAGATGGAAACCGATGCCC-3’ |
| *FGF17* | F:5’-CTGCTGATTCTCTGCTGTCAA-3’ |
|  | R:5’-GTAGAGTTGGTACTCGCGGAT-3’ |

**Information of antibodies**

| Antibodies | Sources |
| --- | --- |
| Tubulin | Wanleibio (Shengyang, Liaoning, china) |
| β-actin | Wanleibio (Shengyang, Liaoning, china) |
| GLUT1 | Wanleibio (Shengyang, Liaoning, china) |
| GLUT3 | Wanleibio (Shengyang, Liaoning, china) |
| HK1 | Beyotime (Beijing, China) |
| LDHA | Wanleibio (Shengyang, Liaoning, china) |
| HIF-1α | Wanleibio (Shengyang, Liaoning, china) |
| pFGFR1(Tyr653/654) | CST (Danvers, Mass, USA) |
| FGFR1 | CST (Danvers, Mass, USA) |
| STING | Beyotime (Beijing, China) |
| Antibodies | Sources |
| pTBK1/NAK (ser172) | Beyotime (Beijing, China) |
| TBK1 | Beyotime (Beijing, China) |
| pIRF3(ser396) | Beyotime (Beijing, China) |
| IRF3 | Beyotime (Beijing, China) |
| α-SMA | Abclonal (Wuhan, Hubei, China) |
| HLA-DRA | Proteintech (Chicago, Illinois, USA) |
| REST | Proteintech (Chicago, Illinois, USA) |
| CD56 | Proteintech (Chicago, Illinois, USA) |
| IFN-α | Wanleibio (Shengyang, Liaoning, china) |
| IFN-β | Beyotime (Beijing, China) |
| HRP-conjugated goat anti-Rabbit IgG (H+L) | Wanleibio (Shengyang, Liaoning, china) |
| HRP-conjugated goat anti-Mouse IgG (H+L) | Beyotime (Beijing, China) |

Reference

1. Lu Y, Li H, Zhao P, et al. Dynamic phenotypic reprogramming and chemoresistance induced by lung fibroblasts in small cell lung cancer. Sci Rep. 2024; 14(1)**:** 2884. https://doi.org/10.1038/s41598-024-52687-z.
